# Effective Melanoma Immunotherapy in Mice by the Skin-Depigmenting Agent Monobenzone and the Adjuvants Imiquimod and CpG

**DOI:** 10.1371/journal.pone.0010626

**Published:** 2010-05-13

**Authors:** Jasper G. van den Boorn, Debby Konijnenberg, Esther P. M. Tjin, Daisy I. Picavet, Nico J. Meeuwenoord, Dmitri V. Filippov, J. P. Wietze van der Veen, Jan D. Bos, Cornelis J. M. Melief, Rosalie M. Luiten

**Affiliations:** 1 Department of Dermatology and the Netherlands Institute for Pigment Disorders, Academic Medical Center, University of Amsterdam, Amsterdam, The Netherlands; 2 Leiden Institute of Chemistry, Leiden University, Leiden, The Netherlands; 3 Department of Immunohematology and Blood Transfusion, Leiden University Medical Center, Leiden, The Netherlands; 4 Department of Dermatology, Netherlands Cancer Institute – Antoni van Leeuwenhoek Hospital, Amsterdam, The Netherlands; New York University, United States of America

## Abstract

**Background:**

Presently melanoma still lacks adequate treatment options for metastatic disease. While melanoma is exceptionally challenging to standard regimens, it is suited for treatment with immunotherapy based on its immunogenicity. Since treatment-related skin depigmentation is considered a favourable prognostic sign during melanoma intervention, we here aimed at the reverse approach of directly inducing vitiligo as a shortcut to effective anti-melanoma immunity.

**Methodology and Principal Findings:**

We developed an effective and simple to use form of immunotherapy by combining the topical skin-bleaching agent monobenzone with immune-stimulatory imiquimod cream and cytosine-guanine oligodeoxynucleotides (CpG) injections (MIC therapy). This powerful new approach promptly induced a melanoma antigen-specific immune response, which abolished subcutaneous B16.F10 melanoma growth in up to 85% of C57BL/6 mice. Importantly, this regimen induced over 100 days of tumor-free survival in up to 60% of the mice, and forcefully suppressed tumor growth upon re-challenge either 65- or 165 days after MIC treatment cessation.

**Conclusions:**

MIC therapy is effective in eradicating melanoma, by vigilantly incorporating NK-, B- and T cells in its therapeutic effect. Based on these results, the MIC regimen presents a high-yield, low-cost and simple therapy, readily applicable in the clinic.

## Introduction

Melanoma patients could benefit greatly from immunotherapy, since melanoma is one of the most immunogenic tumors [1] and metastatic disease responds poorly to conventional therapy, such as irradiation and chemotherapy [2]. Cancer immunotherapy underwent considerable progress in recent years, since the first promising results of adjuvant immune stimulation using interferon-α (IFN-α) and interleukin-2 (IL-2) [3]–[6]. Recent immunotherapeutic vaccination strategies have appeared moderately effective in achieving superior clinical results than standard interventions [7]–[9]. Nonetheless, studies using the toll-like receptor (TLR) ligand cytosine-guanine oligodeoxynucleotides (CpG) as a TLR9 agonist or imiquimod as a TLR7 agonist in the melanoma setting [10]–[17], have shown encouraging results. Successful melanoma immunotherapy can lead to treatment-related vitiligo-like leukoderma as an autoimmune side-effect [18], which is considered an encouraging prognostic sign [19], [20]. Therefore, as a reverse approach, we here investigated the active induction of vitiligo as an immunotherapy approach for melanoma treatment.

Skin contact with phenols or catechols, such as the monobenzylether of hydroquinone (MBEH or monobenzone), induces depigmentation in susceptible individuals upon occupational exposure, which is clinically and histologically indistinguishable from vitiligo vulgaris [21]–[24]. Monobenzone is the most potent skin depigmenting agent [21], discovered by Oliver *et al.* in 1939 [23]. In healthy individuals who have applied it to initially lighten their skin tone it is known to induce vitiligo vulgaris [25]–[27]. Moreover, it has been used in a 20% cream for patients with vitiligo universalis to induce complete depigmentation [27]. The skin depigmentation spreads to distant sites unexposed to monobenzone, indicating that monobenzone induces a progressive systemic reaction against melanocytes, by acting as a skin sensitizer [26], [28], [29]. Monobenzone specifically interacts with tyrosinase [21], [30], the key enzyme in melanocyte pigment synthesis, and forms quinone-haptens to the tyrosinase protein [31]. Quinone metabolites of phenols or catechols have been shown to induce extensive depigmentation *in vivo* [32], [33] depending on the enzymatic conversion by tyrosinase, and covalent binding as a hapten to proteins [30], [31].

Since we have previously shown that vitiligo vulgaris is mediated by melanocyte antigen-specific CD8+ T cells [34], we postulate that monobenzone by its selective interaction with melanocytes, induces melanocyte-specific autoimmunity. In this report we combined the topical skin-bleaching agent monobenzone with immune-stimulating TLR7-agonist imiquimod and the TLR9-agonist CpG [35], [36], designated as MIC-treatment. This combination proved to provoke a robust melanocyte antigen-specific autoimmune response in C57BL/6 *wildtype* mice. This activated response effectively abolished the growth of subcutaneous B16.F10 melanoma. Importantly, the therapeutic effect was found in up to 85% of the mice, while it also mediated over 100 day tumor-free survival in 60% of the mice on average. Innate and adaptive immunity cooperated in the observed therapeutic effect. Our MIC therapy namely induced a melanocyte antigen-specific CD8+ T cell response, a B16-specific serum IgG response and a sustained NK cell expansion. Furthermore, the MIC treatment conferred melanocyte antigen-specific CD8+ T cell-mediated immunological memory that forcibly suppressed secondary tumor growth. Our data establish the MIC therapy as an effective new regimen in the field of melanoma immunotherapy.

## Results

### Expansion and activation of melanoma-reactive CD8+ T cells and NK cells in response to monobenzone, imiquimod and CpG treatment of subcutaneous B16.F10 melanoma

To characterize the *in vivo* immune response induced by monobenzone and the immunostimulatory adjuvants CpG and imiquimod against the highly aggressive and poorly immunogenic B16.F10 melanoma, we inoculated C57BL/6 *wildtype* mice with 2.5×10^3^ B16.F10 cells subcutaneously in the right flank at day 0 (n = 5 mice/group), and from day 2 treated these mice with monobenzone alone, the immunostimulatory adjuvants CpG and imiquimod combined (CI) or monobenzone with imiquimod and CpG (MIC). Importantly, tumors were injected in the flank, while topical applications of monobenzone and imiquimod were selectively applied on the shaved abdomen of the mice; CpG was injected peritumorally. On treatment day 18, mice were sacrificed and splenocytes were *ex vivo* tested for their specific recognition of B16.F10 melanoma. Syngeneic EL4 mouse thymoma cells were used as control.

**Figure 1:**
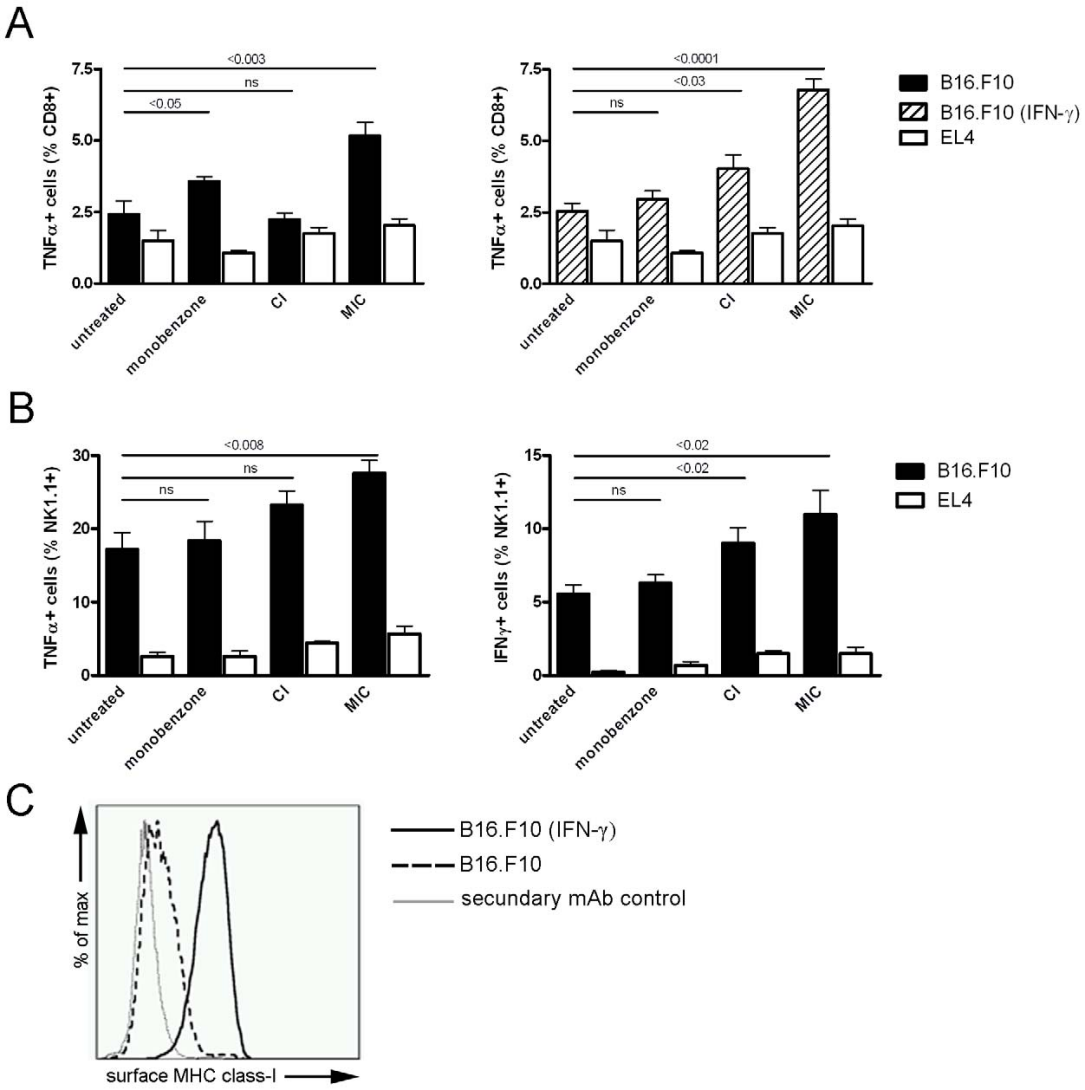
MIC treatment of subcutaneous B16.F10 melanoma induced melanoma-reactive CD8+ T cells and -NK cells *in vivo*. Splenocytes were tested for their *ex vivo* activation upon co-culture with B16.F10 melanoma or EL4 thymoma control cells (n = 5 mice per group). **A**, *Left panel*: CD8+ T cells from monobenzone- and MIC-treated mice showed significant TNF-α production upon co-culture with melanoma cells (*black bars*; p<0.05 and p<0.003 respectively). In contrast, CD8+ T cells from CI-treated mice did not display significant TNF-α production upon melanoma cell co-culture (non significant difference: ns). *Right panel*: To identify CD8+ T cell activation upon co-culture with immunogenic melanoma cells with high MHC class-I expression, co-cultures with IFN-γ primed B16.F10 cells were included (*dashed bars*). Under these conditions, CI-treated mice showed significant TNF-α production as compared to untreated mice (p<0.03). The MIC-treated mice showed even more TNF-α production as in the non-IFN-γ-primed stimulation shown in the left panel. Monobenzone-treated and untreated mice did not display this increased T cell activation upon splenocyte co-culture with IFN-γ primed melanoma cells. T cell activation upon splenocyte co-culture with syngeneic EL4 thymoma control cells showed comparable background levels in all groups (white bars). **B**, *Left panel*: Only NK cells from MIC-treated mice showed significantly increased TNF-α production upon co-culture with melanoma cells (p<0.008). *Right panel*: Elevated production of IFN-γ was found in NK cells from CI- and MIC-treated mice in response to co-culture with melanoma cells (p<0.02 and p<0.02 respectively). TNF-α and IFN-γ production by NK cells was comparable in all groups upon co-culture with EL4 control cells. For the statistical analysis of the *in vivo* tumor growth kinetics of the treatments depicted in this figure, see table 1 (“Exp. 2”). **C**, B16.F10 melanoma cells upregulate their surface MHC class-I expression upon IFN-γ exposure. While IFN-γ-unexposed melanoma cells express very low levels of surface MHC class-I (*dashed line*), priming of these cells with 1000 U/ml IFN-γ restores their surface expression of MHC class-I (*black line*). Control incubations of IFN-γ-primed melanoma cells with only the IgG2a-detecting secondary antibody were negative (*grey line*).

As shown in figure 1A (*left panel*), MIC-treated mice showed significantly elevated percentages of CD8+ T cells producing TNF-α upon recognition of B16.F10 cells *in vitro*, as compared to untreated mice. CD8+ T cells from MIC-treated mice did not react to with the EL-4 control tumor cells, indicating the melanoma specificity of the T cell response. This T cell activation, albeit at a lower level, was also found in mice that were treated with monobenzone alone, while CI-treated mice did not show specific T cell reactivity against B16.F10 cells. Additionally, when B16.F10 target cells were interferon-γ (IFN-γ)-primed to raise their surface class-I expression (figure 1C), making them an optimal CD8+ T cell target, more CD8+ T cell activation was seen in the MIC-treated mice (figure 1A, *right panel*). The IFN-γ primed melanoma cells also evoked T cell activation in the CI-treated mice, while T cell activation was not detected in the untreated- and monobenzone-treated mice. The CD4+ T cell population did not display any significant responses (data not shown).

NK cell activity against B16.F10 melanoma cells was found in all treated mice as well as in untreated mice, as illustrated by TNF-α and IFN-γ production upon co-culture (figure 1B). These NK cells did not react with EL4 control cells, illustrating their melanoma-specific reactivity. However, NK cell activation was significantly increased in MIC-treated mice as compared to untreated mice, as indicated by the production of both TNF-α and IFN-γ. The other treatments did not enhance NK cell activity against B16.F10 cells, except for the increased IFN-γ production by NK cells from CI-treated mice.

Taken together, these *ex vivo* analyses demonstrate that MIC- and to a lesser extent CI-treatment induced the activation of TNF-α-producing melanoma reactive CD8+ T cells. Furthermore, T cells induced by the MIC treatment were also able to recognize non-IFN-γ-primed, poorly immunogenic melanoma cells. This indicates that the MIC treatment generates a significant melanoma-reactive CD8+ T cell population with elevated functional avidity. Moreover, the MIC regimen significantly increased NK-cell melanoma-reactivity *in vivo*.

### Melanoma-reactive IgG response in MIC-treated mice

**Figure 2:**
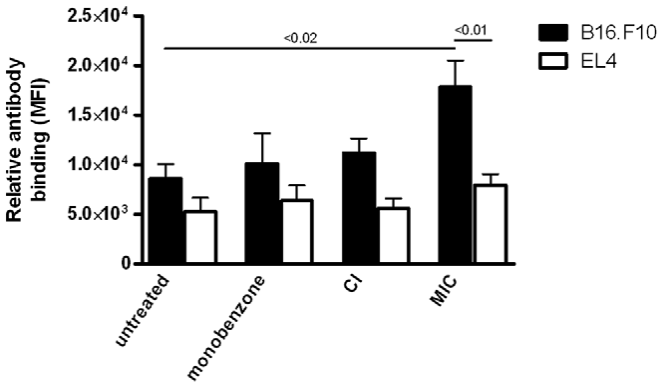
A melanoma-reactive serum IgG response was found in MIC-treated mice. Upon sacrifice peripheral blood serum was obtained from treated mice, and serum antibody binding to B16.F10 melanoma cells was analyzed using flow cytometry (n = 5 mice per group). EL4 syngeneic thymoma cells were used as a control to verify the melanoma-specificity of the antibody binding, and showed comparable serum IgG binding levels in all groups (p>0.05, one-way ANOVA). A significant level of IgG antibody binding to the melanoma cells above the EL4 background level was found in the MIC-treated mice (p<0.01, paired t-test). Furthermore, only the MIC-treated mice showed melanoma-reactive IgG levels significantly above those found in untreated mice (p<0.02, unpaired t-test). IgA controls were negative, and IgM antibodies showed no significant binding levels (data not shown). Data is representative of three independent *in vivo* experiments.

To further characterize the extent of the melanoma-specific immune activation induced by the MIC treatment, we investigated the formation of a melanoma-reactive antibody response. Of the mice described in figure 1, peripheral blood serum was obtained on day 18 of sacrifice. B16.F10 melanoma cells were permeabilized and binding of serum IgG-, IgM-and IgA was determined. Permeabilized EL4 thymoma cells were included as controls. As shown in figure 2, only the MIC-treated mice showed significant levels of melanoma-reactive serum IgG antibodies, whereas no IgG binding to melanoma cells was found in monobenzone- and CI-treated mice. Mice from all tested groups displayed comparable background reactivity to EL4 thymoma cells, illustrating the melanoma-specificity of the IgG-reactivity. IgG binding to intact, unpermeabilized melanoma cells was negligible (data not shown). Melanoma-reactive IgA was not detected and IgM did not show any significant differences between groups (data not shown). The melanoma-reactive IgG response induced by the MIC treatment *in vivo* indicates the involvement of a concurrent B cell response alongside the melanoma antigen-specific T cell activation found in the MIC-treated mice.

### MIC treatment inhibits growth of subcutaneous B16.F10 melanoma

**Figure 3:**
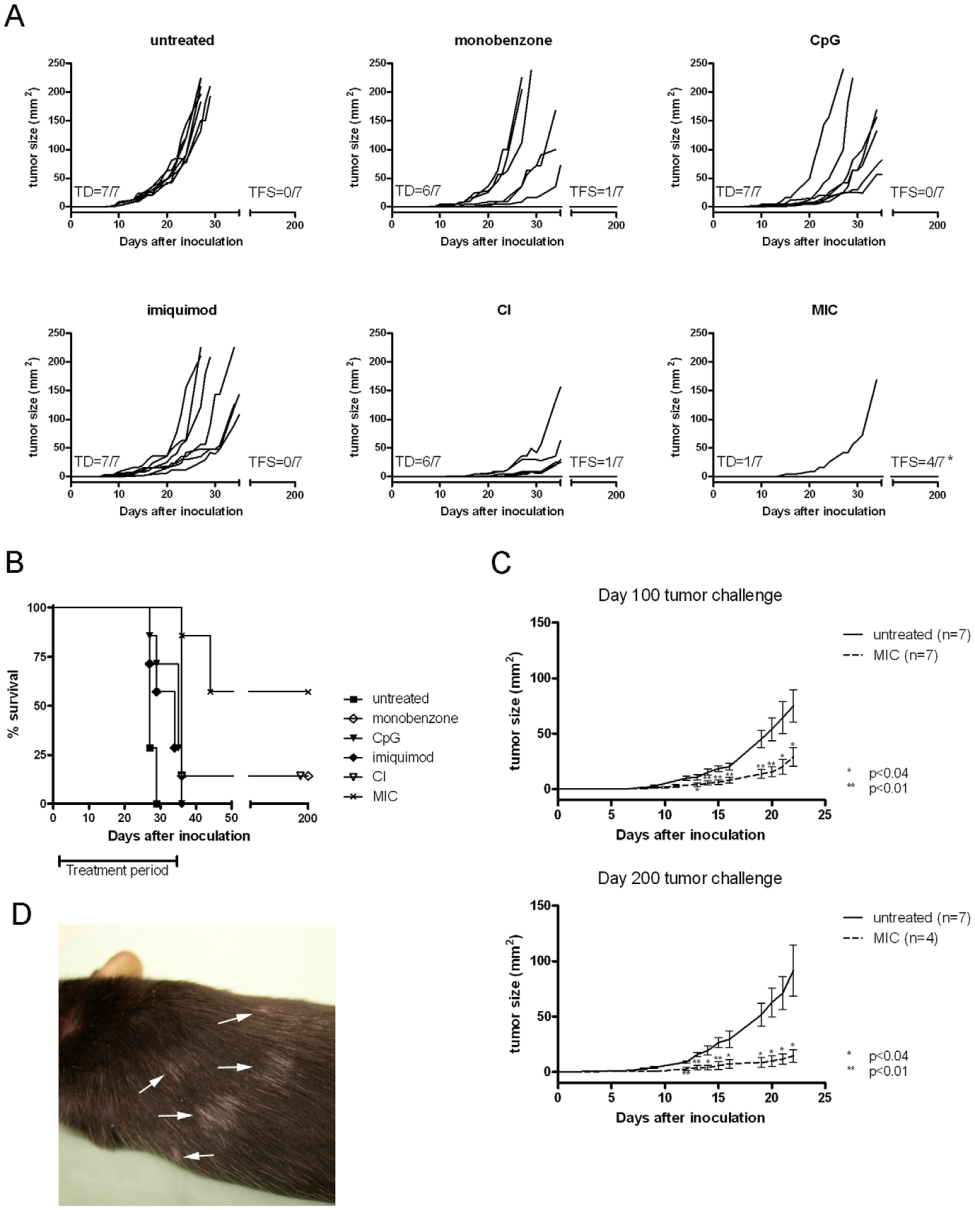
Growth of subcutaneous melanoma is inhibited by MIC therapy. Mice (n = 7 per group) were treated with monobenzone, CpG, imiquimod, CI- or the MIC-regimen. Tumor growth and animal survival were monitored for 200 days. Each graph line depicts an individual tumor growth curve. **A**, *Upper left panel*: Untreated mice all show tumor development (TD) around day 10, and none of the mice experienced a 200-day tumor-free survival (TFS). In contrast, 6 out of 7 MIC-treated mice remained tumor-free during treatment, and only one mouse showed delayed TD during the treatment (*lower right panel*). *:10 days following treatment cessation at day 35, two additional mice developed a melanoma and these animals were directly sacrificed for analysis without monitoring tumor growth kinetics. Eventually, 4 out of 7 MIC-treated mice showed 200-day TFS, since 2 of the mice developed a melanoma 10 days following treatment cessation at day 35. The individual treatment components monobenzone, CpG or imiquimod all mediated a certain degree of tumor-growth delay. Interestingly, CpG and imiquimod clearly work synergistically in the CI regimen. Depicted tumor growth kinetics are representative of 4 independent *in vivo* experiments. For the statistical analysis of the *in vivo* tumor growth kinetics of the treatments depicted in this figure, see table 1 (“Exp. 4”). **B**, Kaplan-Meier survival curve for the different treatment groups depicted under A, showing 57% 200-day survival for MIC-treated mice against 14% long-term survival for monobenzone- or CI treated mice. Mice left untreated or receiving one of the individual treatment components, no TFS was found. These animals were sacrificed around day 25–35 due to maximally allowed tumor burden. **C**, The MIC therapy induced an effective immunological memory response. *Upper panel*: At day 100 (day 65 after treatment cessation) surviving mice were challenged with a melanoma tumor-inoculation, and tumor growth was monitored without further treatment. Untreated naive control mice showed rapid tumor development (*black line*, n = 7). Mice treated previously with MIC therapy showed significant tumor growth delay (*dashed line*, n = 7). *Lower panel*: Mice challenged at day 200 (day 165 after treatment cessation) show protective immunity to a comparable degree to that found in the day 100 tumor challenge shown in the upper panel. Untreated control mice showed normal tumor development (*black line*, n = 7) while mice treated previously with the MIC therapy display significant tumor growth retardation (*dashed line*, n = 4). Depicted graphs represent two independent tumor challenge experiments (follow-up on Exp. 3 & 4 in table 1). Statistical analysis using unpaired t-test comparing MIC-treated mice with untreated animals on designated time points (*: p<0.04, **:p<0.01). **D**, MIC-treated, long-term surviving mice occasionally develop progressively depigmenting patches of fur in a vitiligo-like pattern, distant from the initial monobenzone application site (arrows). This effect occurs in approximately 50% of the long-term surviving animals.

To determine whether the melanoma antigen-specific immune responses described above were able to eradicate B16.F10 melanoma *in vivo*, we treated mice bearing subcutaneous melanoma and performed a long-term follow-up (figure 3A). On day 0, C57BL/6 *wildtype* mice were inoculated subcutaneously with 2.5×10^3^ B16.F10 melanoma cells. Treatments were started on day 2, and continued for 33 days. Mice were either left untreated, treated with the individual treatment components monobenzone, CpG or imiquimod, or with CI- or MIC-treatment regimens and tumor growth was monitored (figure 3A, n = 7 per group). All untreated mice showed tumor development (TD) around day 10, and none of these mice experienced a 200-day tumor-free survival (TFS). In contrast, in 85% of mice treated with MIC therapy the tumor did not grow during the treatment period (figure 3A *lower right panel*). Only one mouse did not respond to the MIC treatment and experienced a (delayed) tumor outgrowth during the treatment period, while all other mice remained tumor-free. 10 days after treatment cessation at day 35, two additional MIC-treated mice developed a melanoma. These animals were directly sacrificed for analysis without monitoring their tumor growth kinetics. This illustrates that MIC treatment effectively suppressed melanoma growth in these two mice during treatment, and that MIC therapy actively induces anti-tumor reactivity without merely preventing tumor implantation. Ultimately, the remaining 57% of the MIC-treated mice experienced tumor-free survival for more than 200 days. The individual treatment components monobenzone, CpG or imiquimod all mediated a certain degree of tumor growth delay, resulting in suppression of melanoma outgrowth in these groups. Furthermore, the combination of CpG and imiquimod acted synergistically in delaying melanoma outgrowth and inducing tumor-free survival. The survival data are summarized in figure 3B, showing the long-term tumor-free survival data of the different treatments. The therapeutic effect of the MIC treatment was found in four independent *in vivo* experiments, which all showed significant inhibition of tumor growth (table 1).

**Table 1:** Statistics of *in vivo* tumor experiments.

			Days after	Average	p-value	TFS during treatment	Median	p-value	TFS >100 days
Exp.	Treatment	# mice	Inoculation^1^	tumor size (mm2)	tumor size*	(# mice)	survival	survival**	(# mice)
				(+/− SEM)	(vs. untreated)	(% of group)	(days)		(% of group)
**1**	untreated	7	22	64.8 (14.3)	nt	0	nt	nt	nt
	monobenzone	7	22	27.1 (7.5)	<0.04	0	nt	nt	nt
	MIC	7	22	3.1 (1.9)	<0.0011	4 (57%)	nt	nt	nt
**2**	untreated	5	18	70.6 (18.0)	nt	0	nt	nt	nt
	monobenzone	5	18	50.0 (29.7)	ns	0	nt	nt	nt
	CpG	5	18	14.4 (3.5)	<0.02	0	nt	nt	nt
	imiquimod	5	18	61.6 (7.2)	ns	0	nt	nt	nt
	CI	5	18	9.0 (4.5)	<0.02	0	nt	nt	nt
	MIC	5	18	5.6 (3.0)	<0.008	2 (40%)	nt	nt	nt
**3**	untreated	11	27	81.6 (20.2)	nt	0	40	nt	0
	MIC	11	27	0.4 (0.2)	<0.0006	8 (72%)	>100	<0.0001	7 (64%)
**4**	untreated	7	27	187.9 (13.4)	nt	0	27	nt	0
	monobenzone	7	27	88.9 (35.3)	<0.03	1 (14%)	34	<0.03	1 (14%)
	CpG	7	27	71.1 (30.0)	<0.004	0	35	<0.005	0
	imiquimod	7	27	102.9 (31.9)	<0.04	0	34	<0.03	0
	CI	7	27	13.0 (5.3)	<0.0001	1 (14%)	36	<0.0003	1 (14%)
	MIC	7	27	6.0 (6.0)	<0.0001	6 (85%)	>200	<0.0003	4 (57%)

ns: not significant (considered if p>0.05). Exp.: experiment.

nt: not tested. TFS: tumor-free survival.

*: Unpaired *t*-test. CI: CpG & imiquimod.

**: Logrank test for survival (endpoint tumor size max 200 mm^2^). MIC: monobenzone, imiquimod & CpG.

1 :Day of tumor size comparison (last day on which experimental animals were all alive).

For Exp. 2 see Fig. 1A/B, for Exp. 3 see Fig. 3C (upper panel), for Exp. 4 see Fig. 3A/B and C (lower panel),

To investigate whether long-term surviving MIC-treated mice developed protective immunological memory, MIC-treated 100- and 200-day surviving mice from two independent experiments were challenged with B16.F10 melanoma cells subcutaneously in the flank. Without further treatment, tumor outgrowth was monitored and compared to untreated, naïve mice. Untreated mice showed comparable tumor outgrowth as seen in figure 3A (*upper left panel*). The MIC long-term survivors all showed a significant growth retardation following the tumor challenge (figure 3C, *upper and lower panel*). These data show that the MIC treatment had induced melanoma antigen-specific immunological memory which remained effective at 65- and 165 days after treatment cessation. Occasionally the development of vitiligo-like patches of depigmented fur at sites distant from initial monobenzone application was found in about 50% of long-term surviving mice (figure 3D). Importantly, vitiligo development occurred exclusively in mice treated with MIC therapy, and was absent in control-treated animals.

As summarized in table 1, the MIC regimen can mediate significant tumor-growth delay and increase tumor-free survival. Although monobenzone, CpG, imiquimod or the CI combination induced significant melanoma growth inhibition, they did not confer long-term tumor-free survival as found in the MIC therapy-treated mice. Combined with the immunological data described in figures 1 and 2, these results indicate that the immunological impact of the MIC regimen translates to significant tumor eradication *in vivo*.

### Activation of an NK cell response in MIC treated mice *in vivo*

**Figure 4:**
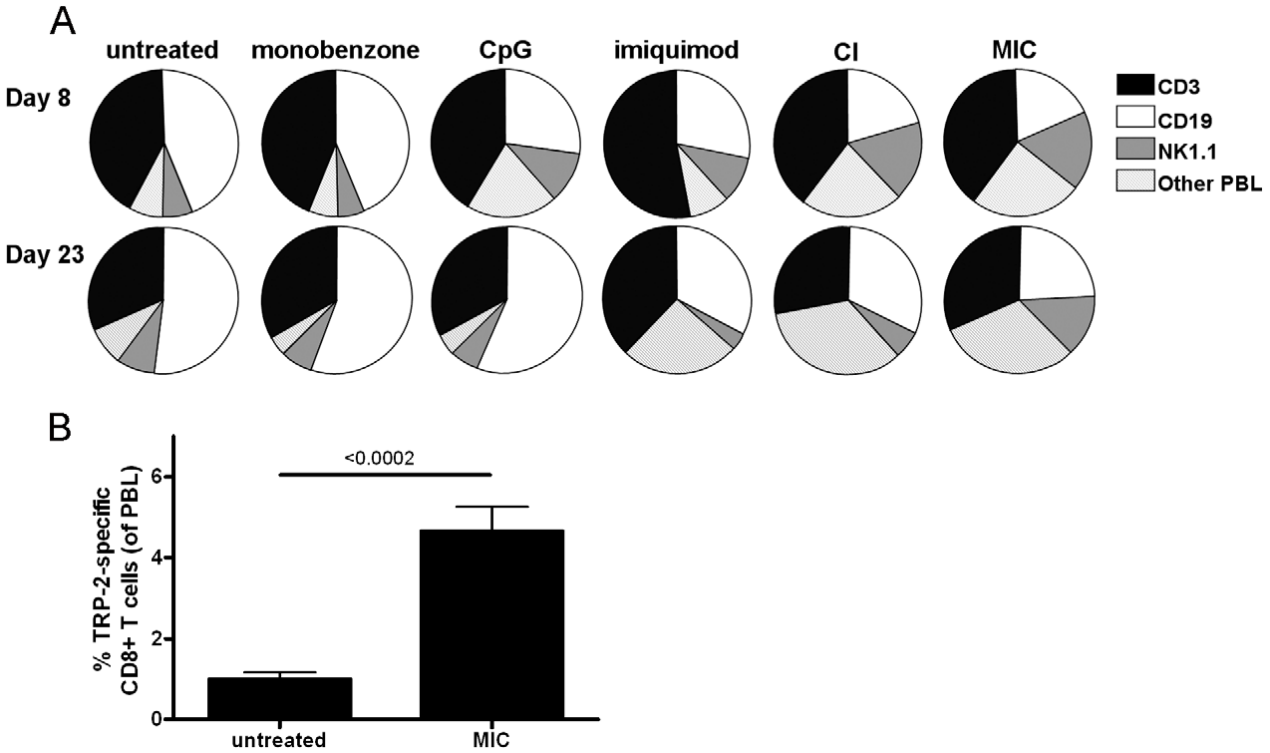
MIC-treated mice show a sustained NK cell expansion and circulating melanocyte antigen-specific CD8+ T cells. **A**, Peripheral blood was collected from the tailvein of treated mice on day 8 and 23 of treatment, and average ratios between T cells (CD3+, black sections), B cells (CD19+, white sections), NK cells (NK1.1+, CD3-, grey sections) and other peripheral blood leukocytes (PBL; dashed sections) were determined for the PBL (n = 7 mice per group). Interestingly, MIC-treated mice showed a significant NK cell expansion on both day 8 and 23 (see table 2, “Exp. 4”). This expansion of NK cells was also found in CpG-, imiquimod- and CI-treated mice, although for these animals this reaction was only found on day 8. Monobenzone alone did not influence PBL ratios on either time point, comparable to untreated mice. Depicted data is representative of three independent *in vivo* mouse experiments. For the statistical analysis of the *in vivo* differences in NK cell counts in these experiments see table 2. **B**, Peripheral blood CD8+ T cells were tested for binding to H2-K_b_/TRP-2_180-188_-tetramers at day 120 following tumor inoculation (day 85 after treatment cessation, n = 4). TRP-2 represents one of the immunodominant epitopes of B16.F10 melanoma. Long-term surviving, MIC-treated mice showed a significant population of TRP-2-specific CD8+ T cells circulating in their peripheral blood at day 120, as compared to untreated mice 10 days after tumor inoculation (n = 7). Binding to control H2-K_b_/OVA_257-264_-tetramer by the tested PBL was negative (data not shown).

We subsequently carried out immune monitoring of treated mice to investigate the *in vivo* immune activation. We determined the ratios of different peripheral blood leukocyte (PBL) populations in blood obtained from the tailvein on day 8 and 23 following melanoma inoculation (figure 4A). Interestingly, the MIC-treated mice (*right panels*) showed increased levels of NK cells (NK1.1+ CD3-) among their PBL on day 8, as compared to the untreated group. NK cell counts were also significantly increased in mice treated with CpG, imiquimod or the combination of these compounds (CI). Importantly, the increased NK cell numbers only persisted up to day 23 in MIC-treated mice (figure 4A). This MIC treatment-induced NK cell expansion has been found in three independent experiments, the statistical analysis of which is shown in table 2. Remarkably, monobenzone alone did not influence PBL ratios at either time point. Taken together, although the CI- and MIC treatment both mediated a significant increase in blood NK cell counts, this effect persisted only in MIC-treated mice.

**Table 2:** Statistics of *in vivo* peripheral blood NK cell counts.

			Days after	average %	
Exp.	Treatment	# mice	Inoculation^1^	NK1.1+/CD3- PBL	p-value*
				(+/− SEM)	
**2**	untreated	5	15	7.3 (0.4)	nt
	monobenzone	5	15	6.3 (0.2)	ns
	CpG	5	15	8.2 (1.1)	ns
	imiquimod	5	15	8.2 (0.3)	ns
	CI	5	15	12.3 (1.9)	<0.04
	MIC	5	15	13.3 (2.2)	<0.03
**4**	untreated	7	8	6.65 (0.7)	nt
	monobenzone	7	8	5.91 (0.2)	ns
	CpG	7	8	11.42 (1.5)	<0.02
	imiquimod	7	8	9.91 (0.7)	<0.01
	CI	7	8	17.29 (1.0)	<0.0001
	MIC	7	8	17.43 (1.6)	<0.0001
	untreated	7	23	8.38 (1.2)	nt
	monobenzone	7	23	7.03 (0.7)	ns
	CpG	7	23	6.17 (0.7)	ns
	imiquimod	7	23	3.81 (0.1)	ns
	CI	7	23	5.95 (0.5)	ns
	MIC	7	23	13.74 (0.7)	<0.01
**7**	untreated	7	2	6.7 (0.4)	nt
	MIC	7	2	16.6 (2.2)	<0.001
	untreated	7	8	4.9 (0.3)	nt
	MIC	7	8	12.6 (1.1)	<0.0001
	untreated	7	15	5.9 (0.8)	nt
	MIC	7	15	12.8 (0.3)	<0.002

Exp.: experiment. CI: CpG, imiquimod.

nt: not tested. MIC: monobenzone, imiquimod & CpG.

ns: not significant (considered if p>0.05). For Exp. 4 see Fig. 4A.

*: Unpaired t-test.

1 :Day after tumor inoculation on which PBL were tested.

Importantly, MIC-treated, tumor-free surviving mice included in tumor challenge experiments (figure 3C) did not display increased blood NK cell counts following the challenge (p>0.05; unpaired t-test at day 15 following inoculation: average percentage 10 (+/− 1.4)). This indicates that the identified NK cell expansion is MIC treatment-dependent. Furthermore, this suggests that the protective immunological memory observed in figure 3C is not dependent on NK cells. Instead, we found circulating melanoma antigen-specific CD8+ T cells in the peripheral blood of these MIC-treated, tumor-free surviving mice (figure 4B). To this end, peripheral blood CD8+ T cells were tested for their recognition of the H2-K_b_/TRP-2_180-188_-antigen. This is an immunodominant CD8+ T cell epitope of B16 melanoma. Figure 4B shows that a significant population of TRP-2 antigen-specific CD8+ T cells is present in tumor-free surviving mice, 120 days following the first melanoma inoculation and 85 days after MIC treatment cessation. Recognition of the control H2-K_b_/OVA_257-264_-tetramer by peripheral blood T cells of these mice was negative (data not shown).

Taken together, these data demonstrate that the MIC treatment effect is likely mediated by activated NK cells and CD8+ T cells. Importantly, when treatment is stopped melanoma antigen-specific CD8+ T cells remain active in tumor-free surviving mice, suggesting that these T cells are responsible for the MIC therapy-induced tumor protective immunological memory.

### CD8+ T cell- and NK cell depletion prior to- and during MIC therapy abrogates therapeutic effect

To verify whether NK cells and/or CD8+ T cells are essential for the therapeutic effect of the MIC therapy, we performed MIC treatment in C57BL/6 *wildtype* mice (n = 5/group) inoculated with B16.F10 melanoma, following either a CD8+ T cell- or NK-cell depleting pre-conditioning monoclonal antibody (mAb) regimen. Control groups either received the MIC treatment following an isotype control mAb regimen, or were left untreated. Depletion of CD8+ T cells and NK-cells was confirmed to be >98% and >95% respectively, as measured by flowcytometry (data not shown).

**Figure 5:**
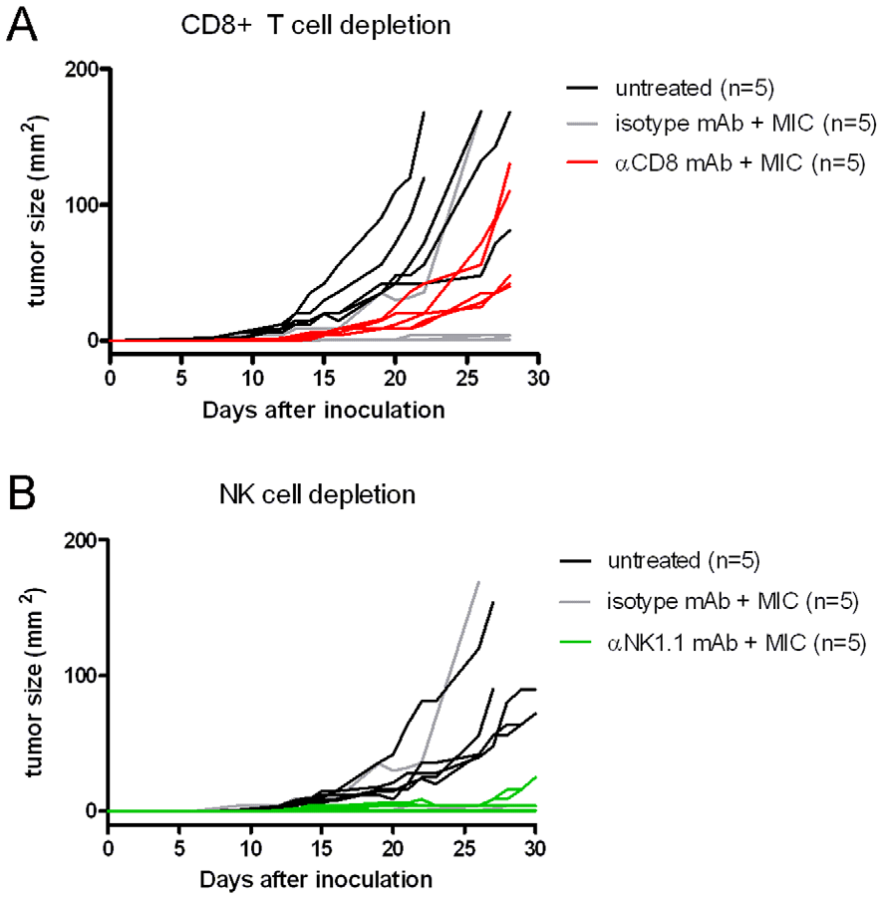
The therapeutic effect of MIC therapy is abrogated by NK cell- or CD8+ T cell depletion. Each graph line represents an individual tumor growth curve. **A**, Mice depleted of CD8+ T cells prior to tumor inoculation and throughout the MIC therapy (*red lines*, n = 5) all developed a tumor. In these MIC-treated mice tumors grew out significantly faster than in the isotype-control mAb-treated mice (*grey lines*; p<0.02, n = 5), who displayed tumor growth kinetics similar to MIC-treated animals in previous experiments. Nonetheless, tumor outgrowth in CD8+ T cell depleted mice was still significantly slower than in untreated mice (*black lines*; p<0.02, n = 5). The tumor size of CD8+ T cell depleted animals was statistically compared with isotype-control mAb-treated animals on day 28, and with untreated animals on day 22. **B**, Mice depleted of NK cells prior to tumor inoculation and throughout MIC therapy (*green lines*, n = 5) showed tumor establishment in 80% of mice. Tumors in these mice grew out very slowly or remained of equal small size throughout the experiment. In contrast, untreated mice showed rapid tumor development (*black lines*, n = 5). Mice treated with isotype-control mAb and MIC therapy (*grey lines*, n = 5) showed tumor growth similar to mice treated with MIC therapy in previous experiments.

As shown in figure 5A, MIC treatment combined with CD8+ T cell-depletion (*red lines*) resulted in melanoma development in 100% of the mice. In contrast, MIC-treated mice receiving isotype control mAb (*grey lines*) showed reduced or no tumor outgrowth comparable to previous experiments (table 1), while melanomas grew out rapidly in untreated mice (*black lines*). Importantly, tumor outgrowth for CD8+ T cell-depleted mice was significantly faster than in mice treated with MIC therapy and the isotype control, nonetheless still significantly slower than in untreated mice. These results show that the anti-tumor effect of the MIC treatment was largely abolished by the depletion of the CD8+ T cells. The minor but significant tumor growth inhibition by the MIC regimen in the absence of CD8+ T cells suggests that this therapy also effectively involves another cell population, likely the NK cells we observed to be activated in previous experiments. Figure 5B shows that depletion of the NK cell population during MIC therapy resulted in tumor development in 80% of the mice (*green lines*). Importantly, melanoma outgrowth rapidly reached a state of “stable disease” in this situation, with tumors remaining of equal size for extensive periods of time. Taken together, these data imply that the MIC therapy effectively engages both CD8+ T cells and NK cells in its protective therapeutic effect.

### MIC therapy significantly suppresses tumor growth of established melanoma

To determine if MIC treatment has a therapeutic effect on larger established melanoma, mice were subcutaneously inoculated with 1×10^5^ B16.F10 melanoma cells and treatments were started when all tumors had reached the minimum measurable size of 2×2 mm (on average after 3–4 days). Mice were treated with the individual treatment compounds alone, or the CI- or MIC regimen. Additionally, combinations of monobenzone with either CpG or –imiquimod as only additional adjuvant were compared with the CI regimen to verify if monobenzone is the critical component in the MIC therapy. The statistical analyses of the tumor growth kinetics in 4 independent *in vivo* experiments using this established tumor setting are summarized in table 3. All treatments containing monobenzone and at least one adjuvant mediated a significant growth delay of established B16.F10 tumors, whereas the adjuvants imiquimod and CpG alone or combined (CI) did not significantly inhibit tumor growth. Of the treatments containing monobenzone, the combination of all three compounds (MIC treatment) showed the most profound therapeutic effect. Importantly, as shown in table 3 under “Exp. 5”, monobenzone combined with either imiquimod or CpG as adjuvants displayed a significantly better therapeutic effect than the CI regimen, when compared to untreated mice. Both exp. 5 and exp. 6 showed that MIC treatment was significantly more effective than CI treatment. This indicates monobenzone to be the pivotal component in mediating the therapeutic effect of the MIC regimen. Besides significantly suppressing tumor growth, MIC therapy was also found to significantly improve the survival of mice bearing established melanoma.

**Table 3:** Statistics of *in vivo* tumor experiments inoculating 1×10e5 melanoma cells and starting treatment when all tumors were at least 2×2 mm.

			Days after	Average	p-value	p-value	Median	p-value
Exp.	Treatment	# mice	inoculation^1^	tumor size (mm^2^)	tumor size*	tumor size*	survival	survival**
				(+/− SEM)	(vs. untreated)	(vs. CI)	(days)	
**5**	untreated	5	15	98.6 (20.7)	nt	nt	15	nt
	monobenzone	5	15	73.6 (10.2)	ns	nt	15	ns
	imiquimod	5	15	74.4 (5.0)	ns	nt	15	ns
	CpG	5	15	55.0 (5.6)	ns	nt	19	<0.05
	CI	5	15	57.8 (8.0)	ns	nt	19	<0.05
	monobenzone, imiquimod	5	15	32.4 (10.3)	<0.03	ns	19	<0.05
	monobenzone, CpG	5	15	32.8 (9.6)	<0.03	ns	22	<0.02
	MIC	5	15	26.4 (7.5)	<0.02	<0.03	22	<0.02
**6**	untreated	3	10	81.3 (29.4)	nt	nt	13	nt
	CI	5	10	1.4 (0.2)	<0.01	nt	28	<0.007
	MIC	5	10	1.0 (0.3)	<0.01	<0.04	24	<0.007
**7**	untreated	7	16	135.6 (24.4)	nt	nt	18	nt
	MIC	7	16	8.9 (3.1)	<0.001	nt	28	<0.002
**8**	untreated	7	16	145.4 (15.1)	nt	nt	16	nt
	MIC	7	16	17.6 (6.3)	<0.0001	nt	26	<0.0001

ns: not significant (considered if p>0.05).

nt: not tested.

1 :Day of tumor size comparison (last day on which experimental animals were all alive).

*: Unpaired *t*-test.

**: Logrank test for survival.

Exp.: experiment.

TFS: tumor-free survival.

CI: CpG, imiquimod.

MIC: monobenzone, imiquimod & CpG.

These data illustrate that the MIC regimen also has a significant therapeutic effect in the treatment of established subcutaneous melanoma.

## Discussion

In the present study we demonstrate that the vitiligo-inducing properties of monobenzone cream combined with the immunostimulatory adjuvants CpG and imiquimod synergistically induces potent melanoma antigen-specific immunity and tumor eradication in the B16-B6 mouse model of melanoma. We show that the MIC treatment of subcutaneous B16.F10 melanoma in C57BL/6 *wildtype* mice inhibited the outgrowth of melanoma in up to 85% of the mice. Moreover, 64% or 57% of the mice remained tumor-free for more than 100- or 200 days respectively. The MIC treatment induced melanoma antigen-specific immunological memory, effectively suppressing tumor growth up to 165 days after treatment cessation. Immunomonitoring of the MIC treated mice showed that the regimen induced a systemic B16-specific CD8+ T cell- and NK cell response. Additionally, a melanoma-specific serum IgG response was found in the MIC treated mice. MIC therapy was also effective against larger established tumors, mediating significant tumor growth inhibition and prolonged survival. These data show that MIC therapy triggers a strong melanoma-specific *in vivo* innate- and adaptive immune response.

It has been suggested by Berghöfer *et al.* that the combination imiquimod and CpG is deleterious, since *in vitro* it has been found that triggering of TLR7 and TLR9 simultaneously on DC or leukocytes will deteriorate the induced interferon-α production by these cells [37]. Importantly, the study by Berghöfer *et al.* concludes that the inhibitory effect of simultaneous TLR7 and -9 triggering only applies to CpG types A and C, while such an effect was not found for CpG type B. Our present study employs CpG 1826, which is a B-type CpG. While Berghöfer *et al.* show that combining B-type CpG with TLR7 triggering was not inhibitory, even showing a slight synergistic trend, we have actually found a functional synergism between CpG 1826 and TLR7-triggering by imiquimod in our present *in vivo* study.

Depletion of CD8+ T cells and NK cells prior to- and during MIC treatment ablated the therapeutic effect, indicating that these cells are imperative in mediating the MIC therapeutic effect. It seems that MIC therapy induces the immune cascade as previously suggested by Liu *et al.* [38], which is characterized by the sequential activation of NK cells, conventional DC and ultimately the formation of effective CD8+ T cell immunity and immunological memory. Importantly, MIC treatment also led to the generation of melanoma-specific antibodies. These antibodies bound to intracellular antigens, selectively in melanoma cells. This indicates they are directed against antigens in the melanosome, an organelle found exclusively in pigmented cells such as melanocytes and melanoma cells. Since the melanosome is the primary source of melanocyte differentiation antigens, the antibodies we identified can enhance the adaptive immune response through opsonisation of antigen liberated from dying pigmented cells. Through the subsequent formation of immune complexes this improves the uptake and successive (cross) presentation of melanoma antigens by DC [39].

Melanoma-specific CD8+ T cells induced by the MIC regimen show a higher functional avidity. As shown in figure 1A, CD8+ T cells in the MIC-treated mice could be activated by non-IFN-γ-primed melanoma cells. These cells have very low levels of MHC class-I (figure 1C), rendering them poorly recognizable to T cells. The occurrence of high avidity T cells recognizing poorly immunogenic melanoma cells suggests that the MIC therapy mediates an avidity maturation process, which is known to occur in T cell populations exposed to constant high levels of antigen [40]. The continuous application of monobenzone on the skin may generate a steady flow of melanocyte-specific antigens, leading to the avidity maturation of the reactive T cell pool. This maturation clearly did not occur during the CI treatment, thereby leading to the absence of high avidity T cells recognizing IFN-γ unprimed melanoma cells in these mice. The CI regimen provides a general immune activating stimulus, which in the presence of melanoma may result in short-term anti-melanoma reactivity. In contrast, the MIC regimen specifically engages melanocyte differentiation antigens in the generation of adaptive immunity via the monobenzone component (discussed below). The therapeutic value of this antigen-targeting by monobenzone is demonstrated by the enhanced ability to induce melanoma-reactive CD8+ T cells and protective anti-melanoma immunity *in vivo*, as compared to the CI regimen.

Concerning the working mechanism of monobenzone, it has a selective- and inactivating interaction with the enzyme tyrosinase which catalyzes pigment synthesis in the melanosome, an organelle exclusively found in melanocytes [21]. This interaction results in the formation of quinone metabolites which bind to cysteine residues in the protein peptide chain, forming quinone-haptens [31]. These quinone hapten-carrier complexes are known to be potent contact sensitizers which can trigger hapten-specific immune responses [41], [42]. Generally, this kind of hapten-induced immunity results in enhanced depigmentation *in vivo*, since quinone-metabolites of phenols and catechols are known to induce more extensive depigmentation than the parental compound [33]. Moreover, the extent of catechol-induced depigmentation depends on quinone formation by the tyrosinase enzyme, including subsequent covalent binding of the quinone to proteins [32]. At high concentrations (250–500 µM for 24 hours) monobenzone can additionally induce non-apoptotic cell death in exposed melanocytes *in vitro* [43]. In contrast, we have investigated that at lower concentrations (20 to 40 µM) monobenzone-exposed pigmented cells provoke robust and specific CD8+ T cell immunity, by the formation of quinone-haptens to the tyrosinase protein and the subsequent activation of local dendritic cells by exposed pigmented cells (Van den Boorn *et al.*, *manuscript in preparation of submission*). Since melanoma cells share many antigens with their normal counterparts, the melanocytes, the melanocyte-specific immunity evoked by monobenzone also acts against melanoma cells (as evidenced by the MIC therapy). Because priming of the anti-melanoma immune response during MIC therapy depends upon the interaction of monobenzone with tyrosinase in skin melanocytes, and the induced immune response encompasses more melanosomal antigens besides tyrosinase (figure 4B and *manuscript in preparation*), our MIC regimen may well be effective against tyrosinase-negative melanoma variants. Moreover, tyrosinase expression appears to be conserved in malignant melanoma cells [44]. Tyrosinase expression in melanoma tissue also provides the possibility of local treatment of cutaneous metastasis with the MIC regimen.

Interestingly, vitiligo-like depigmentation of the fur distant from the monobenzone application site occurred in approximately 50% of the long-term surviving MIC-treated animals (figure 3D), but not in non-responding or control treated mice. This phenomenon illustrates an enduring systemic immune response effective against pigmented cells. However, vitiligo development was expected to directly correlate with the effective anti-tumor immune response observed in all MIC –treated mice, especially since monobenzone is a potent skin depigmenting agent in humans. The vitiligo-like fur depigmentation in mice depends on autoimmune melanocyte destruction in the hair follicle. The modest level of vitiligo may be explained by the fact that hair depigmentation (poliosis) only occurs in advanced cases of vitiligo [45], and is likely related to the hair follicle being an immune-privileged site in both humans [46] and mice [47]. Thereby, fur depigmentation likely underestimates the level of autoimmune activation against pigmented cells. The immune privilege of the hair follicle can thereby explain the modest depigmentation observed in the MIC-treated mice.

In conclusion, we have developed a new form of off-the-shelf melanoma immunotherapy consisting of two creams and four CpG injections. This simple design makes the MIC therapy easily applicable in the clinic. Moreover, this low-cost regimen does not require stringent patient eligibility criteria such as HLA-haplotype, and does not require elaborate patient-specific *in vitro* cell cultures or non-myeloablative lymphodepletion prior to the start of treatment, reducing patient treatment strain.

## Materials and Methods

### Ethics statement

All animal experiments were carried out under protocols approved by the Animal Ethical Committee of the Academic Medical Centre in Amsterdam (DEC code DDE, studies 100452, 100969 and 101291). Mice were obtained from Charles River Labs (Maastricht, The Netherlands). Animals were housed in the Animal Research Institute Amsterdam (ARIA; ABSL-3/DM-II housing level) and cared for by qualified personnel on a daily basis. Food and water were available *ad libitum* and cages contained bedding, shelter and nesting material.

### Cell lines and mice

B16.F10 melanoma cells (a kind gift from Dr. A. Jorritsma, Netherlands Cancer Institute, Amsterdam, The Netherlands) and EL4 thymoma cells (a kind gift from the department of Immunohematology and Blood Transfusion, Leiden University Medical Center, Leiden, The Netherlands) were maintained in culture medium consisting of RPMI 1640 (Cambrex bioscience, Verviers, Belgium) with 10% heat-inactivated fetal bovine serum (Hyclone, Erembodegem-Aalst, Belgium), 2 mM L-glutamine (Gibco invitrogen, Breda, The Netherlands), 50 U ml^−1^ penicillin and 50 µg ml^−1^ streptomycin (Gibco invitrogen), in a humidified atmosphere at 37°C and 5% CO_2_. Female SPF C57BL/6 wildtype mice were used in tumor experiments at 10 weeks of age.

### Tumor inoculation and treatments

Mice were inoculated with 2.5×10^3^ or 1×10^5^ B16.F10 melanoma cells in 50 µl of sterile PBS (Dulbecco's PBS, PAA, Pasching, Austria) subcutaneously in the right flank. Treatments were started 2 days later (for 2.5×10^3^ inoculated melanoma cells) or when all tumors reached a minimum size of 2×2 mm (for 1×10^5^ inoculated melanoma cells on average after 3–4 days). Treatment continued for 33 days. B16.F10 *in vitro* cell culture was standardized for all experiments. Melanoma cells were in the exponential growth phase *in vitro* when used for *in vivo* inoculation, and were confirmed to be >98% viable by trypan blue exclusion. Daily, perpendicular tumor diameters were measured using callipers. Mice were treated with daily applications of 50 µl of 20% monobenzone cream (4-benzyloxyphenol, Sigma-Aldrich, Zwijndrecht, The Netherlands; cream prepared by the Academic Medical Centre pharmacy for use in animal experiments) and/or 50 µl of 5% imiquimod cream (Aldara™, 3 M Healthcare, Leicestershire, UK) on Monday, Wednesday and Friday on the shaved abdomen. Creams were completely massaged in using a spatula. Mice received 50 µl of completely phosphorothioated CpG oligodeoxynucleotides (1 mg ml^−1^ in sterile PBS) injected peritumorally on day 0, 3, 6 and 21 (CpG B 1826, 5′-TCCATGACGTTCCTGACGTT-3′, produced as reported previously [48]).

### In vitro splenocyte priming assay

Erythrocytes were removed from fresh splenocyte single cell suspensions by hypotonic lysis, and splenocytes were cultured in RPMI 1640 (Cambrex) with 10% heat-inactivated fetal bovine serum (Hyclone), 2 mM L-glutamine (Gibco invitrogen), 50 U ml^−1^ penicillin and 50 µg ml^−1^ streptomycin (Gibco invitrogen), 50 mM 2-mercaptoethanol (Sigma-Aldrich, Zwijndrecht, The Netherlands), 15 µg ml^−1^ gentamycin (Duchefa, Haarlem, The Netherlands), 20 U ml^−1^ IL-2 (Novartis, Arnhem, The Netherlands) and 5 µg ml^−1^ ConA (Boehringer Mannheim, Mannheim, Germany). ConA blasts were co-cultured on day 10 with B16.F10 and EL4 cells *in vitro* in a 1∶1 ratio, in culture medium supplemented with IL-2 and 2-mercaptoethanol for 5 hours at 37°C and 5% CO_2_ in the presence of brefeldin-A (1∶1000, Golgiplug, BD Bioscience, San Diego, CA) protein transport inhibitor. When applicable B16.F10 cells were 24 hours IFN-γ pre-treated prior to splenocyte co-culture (1000 U ml^−1^, Strathmann, Bergisch Gladbach, Germany). Subsequently cells were stained for intracellular cytokine production.

### Flow cytometry and intracellular cytokine staining

Erythrocyte-free single cell suspensions of splenocytes, ConA blasts or PBL were prepared in FACS buffer (PBS with 1% bovine serum albumin and 0.05% NaN_3_) and cells were stained in the dark on ice for 20 minutes for surface expression of CD8α (APC-Cy7), CD4 (PerCP-Cy5.5), CD3ε (FITC), CD137 (FITC), CD19 (PerCP-Cy5.5), or NK1.1 (Pe-Cy7; all antibodies BD Bioscience, San Diego, CA). For intracellular staining, cells were permeabilized using the Cytofix/Cytoperm kit (BD Bioscience) according to the manufacturer's protocol, and stained for intracellular cytokines using TNF-α (Pe-Cy7, BD Bioscience), TNF-α (PE, e-Bioscience, San Diego, CA) or IFN-γ (Alexa-700, BD Bioscience). Tetramer analysis was performed by incubating cell suspensions with H2-K_b_/TRP-2_180-188_-tetramer (SVYDFFVWL, APC-labelled, Sanquin, Amsterdam, The Netherlands) or H2-K_b_/OVA_257-264_-tetramer (SIINFEKL, PE-labelled, kind gift from K.L. Franken, Leiden University Medical Centre, The Netherlands) in CM for 25 minutes at 37°C and 5% CO_2_ prior to subsequent surface antibody staining. Surface MHC class-I detection was performed by incubating IFN-γ-primed and –unexposed B16.F10 cells with mouse-anti-mouse MHC class-I antibody (clone 28-14-8, IgG2a, eBioscience, San Diego, CA) followed by incubation with goat-anti-mouse IgG2a-detecting antibody (Alexa 488, Molecular Probes, Invitrogen, Breda, The Netherlands). Samples were measured on a FACS Canto-II flowcytometer (Beckton Dickinson, San Diego, CA). Data was analyzed using FlowJo software (Tree Star Inc., Ashland, OR).

### Serum antibody determination

B16.F10 and EL4 cells were washed in FACS buffer and permeabilized using the Cytofix/Cytoperm kit (BD Bioscience, San Diego, CA) according to the manufacturer's protocol. Cells were incubated with mouse serum diluted 1∶200 in Perm/Wash (BD Bioscience) for 1 hour at room temperature. Subsequently, cells were tested for serum-antibody binding by incubation with biotinylated anti-mouse IgG, -IgA and -IgM antibodies (Biolegend, Uithoorn, The Netherlands) for 20 minutes on ice. Binding of biotinylated detection antibodies to target cells was detected by incubation with streptavidin-APC (BD Pharmingen, San Diego, CA) for 20 minutes in the dark on ice, and analyzed by a FACS Canto-II flowcytometer (Beckton Dickinson, San Diego, CA).

### In vivo NK cell- and CD8+ T cell depletion

Mice were injected intraperitoneally on day -2 and 0 prior to tumor inoculation and every following fourth day during the experiment with 100 µg of anti-mouse CD8α or anti-mouse NK1.1 (clone 53-6.7 or PK136 respectively, low-endotoxin azide-free purified, Biolegend, Uithoorn, The Netherlands) or IgG2a κ isotype control (clone RTK2758, low-endotoxin azide-free purified, Biolegend) monoclonal antibodies. Prior to tumor inoculation CD8+ T cell or NK cell depletion was confirmed by flowcytometry.

### Statistical analysis

Analysis comparing values between treatment groups were performed using a two-tailed unpaired t-test (95% CI). Different conditions within the same treatment group were compared using the two-tailed paired t-test (95% CI). Survival data was analysed using the Logrank test (95% CI). *: p<0.04, **: p<0.01. Analyses carried out with GraphPad Prism 5 software (GraphPad, La Jolla, CA). Graphs depict mean with SEM.
